# The Clinical Society of Maryland

**Published:** 1888-06

**Authors:** 


### Society Reports.

### THE CLINICAL SOCIETY OF MARYLAND.

### Stated Meeting, held March 16, 1888.

The 207th meeting was called to order by the President, Dr. N. G. Keirle in the chair.

Dr. Geo. J. Preston read a paper on

ATAXIC LATERAL SCLEROSIS, AND EXHIBITED THE PATIENT.

Dr. F. T. Miles said that he had seen several cases of this disease in the last two or three years. In one there was the spasmodic condition present with pain, etc., but the generative functions remained intact for a long time. In another case there was great weakness of the lower extremities, but very little spasm there, though the latter symptom was decidedly marked in the abdominal muscles.

Dr. G. J. Preston, in reply to a question from Dr. Branham, said that any voluntary movement on the part of the patient he had shown produced spasm, but no decided tremor could be detected.

Dr. H. Clinton McSherry read the next paper reporting

UNUSUAL SYMPTOMS FOLLOWING THE LOCAL USE OF COCAINE.

Dr. R. H. Thomas reported several additional cases on the same subject.

Dr. N. G. Keirle, in discussing the subject, spoke of organic shock. He said that such symptoms had been observed by gynecologists after applications had been made to the uterus. Manipulation about the throat will sometimes bring on peculiar symptoms, irrespective of what is being used. He related a case in whose throat an application was made, and it brought out such serious symptoms that he feared the trachea would have to be opened before relief could be given.

Dr. Herbert Harlan said that he agreed with Dr. Keirle in his reasoning regarding the effect that manipulation will have on certain locations in the body, but he had never seen any bad symptoms coming on after the use of cocaine locally to parts outside of the throat. Every day it is used in eye practice. A five per cent. solution is dropped into the eye five or six times at short intervals, and no bad symptoms had he ever observed from its use. Subcutaneous injections of 10 to 15 m. of a solution of the same strength have been used for the purpose of removing small tumors, and it has always acted well. There must be some peculiarity about its action when applied to the nasal mucous membrane.

Dr. Samuel Theobald said that he used cocaine every day in his eye practice. Some of it must get into the nasal duct, but he could not recall a single instance of its bad effect. He had used it in his own family for allaying the pain of toothache where some of it must have been absorbed; no bad symptoms ever followed. He thinks that some of the fluid might have gotten into the larynx while being applied, and that probably was the cause of some of the symptoms described by Drs. McSherry and Thomas.

Dr. F. T. Miles said that the symptoms described may have been due to something in the operative procedure. He had used it frequently with children, and also in cases of irritability of the larynx, and had never had the slightest bad results.

Dr. I. E. Atkinson thought that in the vast majority of cases cocaine can be used with entire safety; but a sufficient number is now on record to show that in some an idiosyncrasy exists, and that bad symptoms follow its use. In some few instances death is even the result. Apart from these considerations there is a specific action belonging to the drug which must be remembered.

Dr. W. H. Norris said he had used cocaine in many cases of earache, toothache, etc., and had never seen any ill effect from it. He believed in the idiosyncrasy that some possess to the effect of this drug as well as to other agents. Sometimes the preparation we use may be impure, and this may be the cause of the bad symptoms, if any. He had used it in one case of irritable urethra in a female with the most happy results.

Dr. Hiram Woods spoke of a case he had seen at the Presbyterian Eye and Ear Hospital. The patient was a boy, aged 9 years, who had a foreign body in his eye. For the purpose of removing it a few drops of cocaine were instilled, and the patient was told to sit on a bench till it had its effect. No sooner had he seated himself when it was noticed he had fainted. Water was thrown in his face and he soon came to, but fainted again. After recovering this time cocaine was again distilled, and the body was removed without any other symptoms following. He did not believe this effect was caused by the action of cocaine.

Dr. Samuel T. Earle called attention to the cases he had published, where serious symptoms came from the local use of cocaine in operations performed about the rectum.

Dr. William Rickert related a case of glossitis, where he had used a four per cent. solution of cocaine, and it produced serious symptoms. He was suddenly called to the patient and found him moribund. Restoratives were given and he got better. The cocaine was continued again and the symptoms returned. He then stopped its use, gave stimulants, and the patient did well afterwards. In another case of conjunctivitis cocaine did badly and produced unpleasant symptoms. He used morphia and atropia instead, and all went well.

Dr. Richard Thomas said cocaine was beneficial in the majority of cases, but we should always be on our guard. In regard to one of his cases, where bad symptoms came on, he had made applications with nitrate of silver, and also with weak solutions of cocaine, with no bad effects. It was only when stronger solutions were used that the unpleasant effects were noticed. In the case reported by Dr. McSherry pledgets of cotton, saturated with the solution, were put into the nostrils. He had used other remedies in the same location without producing any bad symptoms; therefore he thinks that this disproves the theory of this special location having anything to do with the effect produced by cocaine.

Dr H. C. McSherry said that unless he had found that the majority of the members present were of a different opinion from himself regarding the action of cocaine, he would never have been led to write this paper. Very few believe in the bad effects of the drug, and for that reason attention should be drawn to it. He did not intend in his paper to go into a general discussion of the effects of cocaine, but simply wanted to point out certain symptoms that may arise when it is applied to the throat. It seems to be impossible that a few drops getting into the larynx could produce such symptoms as those observed in his cases. We should always be careful, though, in our manipulation when applying it.

Dr. L. McLane Tiffany related

CASES OF LAPAROTOMY FOR DISEASES OF THE LIVER.

CASE I. was an hepatic abscess. Patient was a white male, æt. 24 years. In October, 1884, he was taken with an attack of dysentery, which reduced him very much in his general health, but in February, 1885, he had improved and was up and about. In March of the same year he went South. While there dysentery again attacked him, and he returned home in April. In July he went to Deer Park and got better. He went to Newport in August and became worse, and his bowels continued irregular during that winter. In the spring of 1886 his bowels were still irregular, and his physician stated that he had congestion of the liver. During the summer of 1886 the patient had a capricious appetite, lassitude, irregular bowels, and perhaps a chill or so (but this latter uncertain) were noticed. Amelioration in his condition did not occur during the fall. He saw the patient January 20, 1887. Examination of all his organs gave negative results. He was kept under observation and the changes noted during three weeks. In February a diagnosis of liver abscess was made. On March 9th he was tapped, but the contents soon re-formed. An operation was then decided on, and was performed March 19th, 1888. The patient was etherized, and an incision made over the region of the liver below the ribs. Evidence of the abscess being situated in the superior portion of the organ, it was decided to get free drainage from below. The parietal layer of the peritoneum being cut through and the liver reached, it was decided to close off all contact with the peritoneal cavity by stitching this layer firmly to the visceral layer of the liver, which was done with a glover's stitch. The incision was then carried through the liver and the abscess reached. During the incision a good deal of hemorrhage took place, but it was easily controlled by packing some lint into the wound. After it ceased a drainage-tube was inserted and the abscess cavity thoroughly drained. The wound was then united and dressed with iodoform gauze, etc. Early in April bile began to flow through the wound, and so profusely did it do so that it became necessary to withdraw the drainage-tube. In the meantime the patient was given ox gall internally. All symptoms soon subsided, and he went on and made a perfect recovery.

CASE II—*Biliary Calculi.*—This patient was seen in consultation with Dr. Wilkins. A female, æt. 30 years, married and the mother of several children. Previous health good. Since August 1st, 1887, she has had frequent attacks of pain which presented the clinical history of biliary calculi. The paroxysms of pain would recur at intervals of one to two weeks, of variable duration and usually sudden in their onset and disappearance. Jaundice always followed the attacks of pain. The urine showed the presence of bile and the feces were clay-colored. Vomiting usually followed the attacks and throughout dyspeptic symptoms predominated. Palpation and percussion showed pain and tumefaction in the right hypochondriac region. Gall-stones were found from time to time in the feces. After exhausting all of the usual means of treatment operative aid was proposed and accepted by the patient. On the day of the operation the patient was emaciated, skin and conjunctiva considerably icteric. Pulse 96, temperature 99.6.° In the right hypochondriac region a a tumor could be felt which was painful to the touch, movable, and it would change its position toward the median line when the patient was placed on the left side. The operation was performed January 17th, 1888, under strict antiseptic precautions. The patient was etherized and an incision was made over the seat of the tumor. The liver was reached and the hand was introduced through the wound. Examination of the surrounding parts showed the gall bladder to be adherent to the intestines, which interfered considerably with its manipulation. Such being the condition it was decided to cut through the liver and reach the gall bladder that way. The parietal layer of peritoneum was firmly stitched to the visceral layer of the liver, as was done in the case of liver abscess, thus closing completely the peritoneal cavity. An aspirator needle was then inserted through the liver substance towards the gall bladder and a small amount of viscid fluid was seen to come away. A knife was then introduced along the side of the needle and the gall bladder was found and opened. Quite a gush of hemorrhage took place, which was controlled by thrusting the finger into the wound. Fifteen gall-stones varying in size were removed. A drainage-tube was introduced, the wound brought together and dressed with iodoform gauze. The patient soon recovered from the anæsthesia; one grain of opium was given every three hours and from this time all went well towards recovery. January 21st the drainage-tube was removed, and on January 22d the operation showed the presence of bile. Jaundice at this time was disappearing and by January 31st it was almost entirely gone. February 2d the sutures were removed and perfect union had taken place except a small fistulous opening. February 24th the patient had gained in flesh and strength ; all symptoms have subsided and she is gradually resuming her household duties.

DISCUSSION.

Dr. H. Rolando said that he had seen three cases of gall-stone that had been operated on and all were followed by death. One case in particular the operation was done, but it failed. An examination of the parts afterwards showed the gall bladder to be adherent to the pancreas. He thinks if such had been the case in Dr. Tiffany's patient his method of operating would not have been so successful.

Dr. Wm. Rickert said the paper read by Dr. Tiffany was one of the most interesting and important that had been read before the Society this season. It taught an important lesson of what could be done and it afforded a good example for the young surgeons to follow. He hoped that they all might be able to accomplish such results.

Dr. George J. Preston said that he had seen one case of supposed gall-stones some years ago that was under the care of Dr. W. W. Keene. The case was diagnosticated as one of gall-stones with a history of having had them for two years. Dr. Keene cut down on the gall bladder, but no stones were found. Some adhesions had taken place. He soon had to cease operating on account of the condition of the patient. Recovery took place, however, and there was no return of the trouble.

Dr. W. H. Norris said the paper of Dr. Tiffany was a very interesting one to him. It showed the results that could be gotten when one was capable and experienced in such work. He condemned the promiscuous way in which capital operations were being done these days by those who are incompetent to perform them. He had once a case similar to the one reported by Dr. Tiffany, where the patient had suffered for eighteen months from very severe symptoms. The late Prof. Smith was called to see her and he gave but little comfort about her condition. He said it was cancer. She became very much jaundiced. Another physician was called and he decided that it was not cancer and ordered to be given dilute nitric acid in 12-drop doses. She went into the country for awhile; the same treatment was continued when she returned and to-day she is well and hearty.

Dr. Herbert Harlan said that he had seen a liver in the dissecting room in which he thought there had been an abscess. The right lobe was adderent to the viscera; the left one was hypertrophied and the lower half of it was consolidated. There was no evidence of inflammation. He concluded there must have been an abscess and that cicatricial tissue had replaced it. He did not know of what the patient died.

Dr. Randolph Winslow said that all of the cases of liver abscesses he had seen occurred in Germans. One died without operation; the other was operated on, got well, but died six months subsequently from dysentery. The aspirator is invaluable as an aid to diagnosis, but of no use as a remediator. He looked up the literature of liver abscess some years ago and found that in cases where over fifty ounces of pus were drawn off they never got well from aspiration. Operation afterwards was necessary to bring about a cure.

Dr. I. E. Atkinson called attention to the case of liver abscess, aspirated some years ago by the late Dr. Richard McSherry, where over ninety ounces of pus were drawn off and the patient made a perfect recovery.

Dr. L. McLane Tiffany, in reply to a question, said, as regards the diagnosis of the presence of gall-stones, he did not see how it could be made with certainty without opening the bladder. He agreed with Dr. Winslow about the aspirator. It is useful in making a diagnosis of abscess, but almost invariably its use is followed by operation. The nationality of liver abscess is somewhat of a conundrum and is in no way satisfactorily explained.

---

STATED MEETING, HELD APRIL 6, 1888.

The 208th meeting was called to order by the President, Dr. N. G. Keirle in the chair.

The first paper of the evening was read by Dr. W. S. Halstead on the treatment of

CANCER OF THE BREAST.

He also exhibited a number of instruments and microscopical sections.

Dr. O. J. Coskery said the Society ought to feel greatly obliged to Dr. Halstead, not only for the valuable suggestions he had made in his paper, but also for the careful way in which he had prepared his specimens for illustration. He thinks he would infer from the doctor's remarks that the Pectoralis Major is always involved in the cancerous growth, but it is not so. The suggestion to take away a large amount of skin when operating is advisable, though he does not think it the rule to do so. The cause of recurrence of cancer is put down to the fact that all of the growth had not been removed by the operator. He thinks probably that the irritation produced by the incision may be a cause, as the growth frequently recurs in the scar. This recurrence, then, starts in the skin and not in the Pectoralis Major. Consequently the surgeon should not be blamed for leaving some of the growth behind when recurrence here does take place.

Dr. N. G. Keirle said that he could give an example of the early involvement of the Pectoralis Major. In one instance the tumor was small, but the nipple had retracted, due to the Pectoralis Major being adherent to it. The cause of this retraction of the nipple varies—an abscess can produce it by the position which the pus may assume.

Dr. W. S. Halstead said that he did not think that he had said anything to convey the imprssion to any one that the Pectoralis Major is always involved in the growth of cancer. In small growths it is thought not to be so, though an examination does often find it to be involved. It is impossible to see it with the naked eye. He does not agree with Dr. Coskery that cancer will recur if no germs are left. The incision of the old operation leaves an amount of redundant tissue which can be utilized in making the flap which he recommends. In reply to a question whether the removal of the Pectoralis Major impaired the use of the affected side or not, he said the two cases, of which he had a history since operation, could go through all of the motions belonging to the part, but they were not so strong as they were before the muscle was removed.

Dr. F. C. Bressler read the next paper on

ABSCESS OF THE CEREBELLUM FOLLOWING SUPPURATIVE OTITIS MEDIA.

Dr. Hiram Woods said that he is situated so as to see a good deal of this kind of affections. He does not believe that any surgical aid would have done any good in this case. Chronic suppurative otitis may exist with abscess for a long time without giving rise to any trouble. Roosa says they occur more frequently in the cerebrum than elsewhere. In the cerebellum they are very rare. The course of treatment for these affections which he has adopted for sometime corresponds pretty closely to that laid down by Roosa in his book. First recognize that the trouble in the middle ear may give rise to mastoid inflammation. If such seems to be the case, put on six leeches, and if this does not relieve in twenty-four hours, then make Wile's incision, put in a tent and in many cases the trouble will stop there. If not, the whole bone should be examined with a probe, and if any pus is present it will be evacuated by touching the soft points. If this does not relieve, the mastoid should be opened, providing there is no evidence of brain disease. Perri-auricular inflammation may sometimes be mistaken for abscess. He had a case of this kind a few days ago. Leeches were applied, and he thought the trouble would subside, but it did not. The patient was then chloroformed, and Wile's incision made, but it did not relieve the condition. Pain became very intense and large doses of opium were required to alleviate it. His trouble is probably a periostitis. He has no faith in iodoform in these troubles. It has little effect in discharges from the ear. Absolute cleanliness is necessary. If the symptoms point to mastoid trouble, we should lose no time in cutting down upon it, and if pus is present let it out.

Dr. Geo. B. Preston said that this case of Dr. Bressler corresponded very closely with similar ones that have been described. When the middle lobe of the cerebellum is affected it is a different thing and the result is a permanent lesion. All of these cases are interesting from a surgical point of view. While they are not so favorable for operation as affections of the cerebrum, yet they offer some ground for hope.

Dr. Hiram Woods, in reply to a question, said he preferred boracic acid to all other remedies in ear discharges. There are some cases, though, in which it will do very little good, especially if the perforation is small, because it is dificult to apply it to the surface. If the opening is large we can use an insufflator. Alcohol and boracic acid are the best remedies for suppurating ears.

Dr. J. C. Hemmeter called attention to two cases of suppurative otitis recently published, neither of which gave any decided symptoms of its presence.

Dr. J. H. Branham said it was hard to explain the symptoms of paralysis in Dr. Bressler's case. He had once treated a case that came to the City Hospital with symptoms of meningitis. The patient had ear trouble and he was relieved by being bled rather freely.

Dr. F. C. Bressler said that it probably would have been hazardous to have operated in this case, but with such symptoms there was a possibility of its doing good. The post-mortem showed, too, that had they trephined it would have struck exactly the seat of trouble. In regard to the literature on the subject he admitted that he had not fully looked it up and that was the reason he did not know of the reported case referred to by Dr. Hemmeter.

---

### STATED MEETING, HELD APRIL 20, 1888.

Dr. A. B. Arnold reported a case of

### BRAIN DISEASE.

The patient was a girl, aged 14 years, who had come under his care at his clinic at the College of Physicians and Surgeons. She had passed safely through all the diseases of childhood and enjoyed good health up to ten months before her admission into the hospital. At this time she began to suffer with headache, which continued to grow in violence until she came under his care. An examination showed the girl to be small and delicate in appearance. She was blind in the right eye and nearly so in the left one. This condition has been present for nearly two months. She was intelligent and quiet in temperament. When she would walk a swaying of the body took place. Vomiting occasionally came on without apparent cause. With these symptoms of violent headache, vertigo, vomiting and swaying gait, he made the diagnosis of brain tumor and then gave the case to Dr. Friedenwald to be examined with the ophthalmoscope. As regards the location of the tumor, the vomiting, vertigo, etc., would give but little clue to it, but the swaying gait would point to the fact of its being situated at the base of the brain. Still there would be some objection to this, for we ought to expect some paralysis, though there was none whatever. The headache also induced him to suspect that the base was affected. Frequently in children these brain troubles are tubercular in nature and this was so suspected. After being under treatment for six weeks she was taken with coma and died.

Dr. A. Friedenwald spoke of the ophthalmoscopic examination. There was no disturbance of motility. She was blind in one eye and nearly so in the other. There was atrophy of the optic nerves and the question arose, was it primary or secondary? He was disposed to exclude the choked disc because of the dilated and distorted condition of the blood-vessels that was present. He, therefore, concluded that the affection was one of primary atrophy superinduced by pressure.

Dr. J. W. Chambers did the post-mortem examination. The autopsy was done fifteen hours after death. Nutrition fairly good. All of the organs, except the brain, were healthy beyond a few nodules found in the apex of the left lung which proved to be remains of catarrhal pneumonia. There was no evidence of meningitis. At the base of the brain a cystic formation was found which extended backwards and it contained about four ounces of fluid. After removing the brain from the skull, section was made and it was found that the growth was pressing upon the velum interpositum and also encroaching on the chiasm and optic tracts. It was cystic in character and would probably be classed as a psammoma. Whether it was an encysted hydrocephalus is hard to say. Why it did not cause general paralysis is very hard to explain—probably its cystic nature was the reason, as a solid tumor would have been more apt to have done so. The microscope showed around the sheath of the vessels a good many cells, which were probably lymph cells, though some who saw them thought they represented a degree of inflammation.

In reply to a question from Dr. Woods, Dr. Arnold said there was no history of any injury to be gotten from his patient.

Dr. Hiram Woods said about two years ago, at the Presbyterian Eye and Ear Hospital, a girl, aged six years, was brought there to be treated for blindness. Her history showed that in December previous, while playing, she received a fall and struck the back part of her head. This produced convulsions, but she recovered in four or five days from them. After this her eyesight became defective and the ophthalmoscope showed atrophy of both optic nerves. An unfavorable prognosis was accordingly given. She then went to several different physicians and they all gave the same opinion. She subsequently lived for about one year and became totally blind. Convulsions again returned and she finally died. No autopsy could be had. The probable diagnosis was a tumor at the base of the brain. He also had seen a case in the practice of Dr. Caleb Winslow after the patient had recovered from measles. The eyes had failed to some extent and the optic nerves were found beginning atrophy. There was also present some injection of the retinal vessels.

Dr. A. B. Arnold said in these cases blindness is a common symptom. Any brain tumor at the base will cause effusion by pressing on the surrounding parts. Sometimes they are latent and then cause no trouble. Again violent headache may be the only symptom pointing to its location. General symptoms of optic neuritis will cause such evidence of choked disc. Tumors which interfere by pressure on certain parts of the brain will give rise to symptoms of location. There was no motor disturbance in this case because there was no interference with the motor trank. When the growth is slow the parts accommodate themselves to it. The situation of this tumor explains as many of the symptoms as can be possible.

Dr. W. P. Chunn read a paper on rare forms of

ABDOMINAL TUMORS IN THE NEGRO RACE.

Dr. Whitfield Winsey said that in 1881 a case came under his notice in which there was a history of ovarian cyst. The patient was a colored woman, aged 45 years. He confirmed the diagnosis by drawing off some fluid with a hypodermic syringe in which the microscope showed the pressure of cells. The late Dr. Erich saw the case with him and unhesitatingly agreed with him in pronouncing it an ovarian cyst. She died before an operation was performed. To base a diagnosis on the fact of a certain class of tumors being rare in a certain race is unscientific, to say the least. The patient should have the advantage of all of the modes advanced by science in reaching a conclusion. He thinks the paper of Dr. Chunn is valuable inasmuch as it draws our attention to this important point.

Dr. R. M. Hall said that while he had had a good deal of experience in treating diseases in colored women, he had never been able to detect an ovarian tumor. While he was attending college, a colored woman was brought before the class with a tumor which was diagnosticated as ovarian, but when the operation was performed it turned out to be a fibroid of the uterus.

Dr. W. P. Chunn said that he was glad to know that those of experience agreed with him on this subject. It seems strange that something has never been written about it before. He tried to find some literature referring to the matter, but was unable to do so. There is no doubt that if the subject is more investigated more cases will come to light and thus make the diagnosis easier. It is a subject of much interest and importance.

